# Job satisfaction and its associated factors among opticians in Ghana: a cross-sectional study

**DOI:** 10.1186/s12960-021-00612-0

**Published:** 2021-05-17

**Authors:** Kwadwo Owusu Akuffo, Akosua Kesewah Asare, Elsie Emelia Yelbert, Emmanuel Kobia-Acquah, Emmanuel Kofi Addo, Eldad Agyei-Manu, Thomas Brusah, Prince Antwi Asenso

**Affiliations:** 1 Department of Optometry and Visual Science, College of Science, Kwame Nkrumah University of Science and Technology, Kumasi, Ghana; 2 Optical Department, Sight for Africa Eye Clinic, Accra, Ghana; 3 Low Vision Department, Eastern Regional Hospital, Koforidua, Ghana; 4 Department of Ophthalmology and Visual Sciences, University of British Columbia, Vancouver, Ghana; 5 Center for Eye Research Ireland, Technological University Dublin, Dublin, Ireland; 6 Department of Ophthalmology and Visual Sciences, John A. Moran Eye Centre, University of Utah, Salt Lake City, UT USA; 7 Department of Nutrition and Integrative Physiology, University of Utah, Salt Lake City, UT USA; 8 Usher Institute for Population Health Sciences and Informatics, College of Medicine and Veterinary Medicine, University of Edinburgh, Edinburgh, UK

**Keywords:** Dispensing optician, Factors, Ghana, Human resource, Job satisfaction, Opticians

## Abstract

**Background:**

Job satisfaction refers to the feeling of contentment one experiences with their job. Job satisfaction among opticians is a crucial variable in determining their motivation and has consequential influence on the quality of eye health care, systems and services. Nevertheless, little has been done to assess job satisfaction levels among human resources for eye-health, such as opticians, in Ghana. This study assessed (for the first time) the job satisfaction level among opticians in Ghana, and the factors associated with their job satisfaction.

**Methods:**

This was a cross-sectional survey involving all registered and licensed opticians working in Ghana. A validated, well-structured job satisfaction questionnaire was distributed to 195 opticians across all regions of the country. The questionnaire was composed of 15-item job satisfaction variables which were measured on a five-point Likert scale (‘1—strongly disagree’ to ‘5—strongly agree’). Logistic regression analyses were used to investigate the association between sociodemographic characteristics and factors of job satisfaction, and the overall job satisfaction level.

**Results:**

A total of 101 opticians responded to the study. The mean presenting age of all participants was 25.3 ± 5.0 years (21 to 47 years), with majority being males (57.4%). The mean score of the overall job satisfaction level reported by participants was 2.65, with 12.9% (95% confidence interval [CI]: 7.0–21.0%) of them being satisfied with their jobs. There was no statistically significant association between overall job satisfaction and sociodemographic characteristics (p > 0.05; for all). Only salary was significantly associated with overall level of job satisfaction (odds ratio [OR]: 16.5; 95% CI: 2.06–132.86; p = 0.008).

**Conclusion:**

Majority of opticians working in Ghana were not satisfied with their jobs. Enhancing salary/remuneration would improve the job satisfaction level among opticians in the country. There is the need for effective management of human resources for eye-health (particularly opticians) and policy revision on ophthalmic healthcare administration in Ghana.

## Background

The delivery of healthcare services is hinged on three key factors: organizational (health delivery), environmental (infrastructure) and individual (human resource) factors [1]. However, human resource is the most essential component in the provision of high-quality healthcare services. Employee satisfaction has been shown to positively influence efficiency, productivity, and quality of services rendered by organizations [1–3]. Employee satisfaction is thus an excellent indicator of individual well-being, and a strong predictor of staffs’ turnover intentions [4, 5]. It is a critical variable in assessing the productivity of employees, and their perception on responsibilities and motivation.

In order to fully comprehend the concept of job satisfaction, several theories have been proposed. These include, but not limited to, the content theory of work motivation (needs hierarchy [6]; two-factor theory [7]; and the existence, relatedness and growth [ERG] theory [8]), the process theory on cognitive antecedents (expectancy theory [9], Porter–Lawler model [10], and goal-setting theory [11]) and the equity theory on perception of fairness of rewards. Each of these theories aims at explaining the attitude and motivation of employees so they are better understood by managers. Researchers have also tried to define job satisfaction based on these theories. For instance, Sinha [12] describes job satisfaction as contentment derived from engaging in a piece of work (that satisfies one’s need and brings a sense of fulfilment) or an achievement through the pursuit of a higher calling. It is fundamentally related to human needs and the fulfilment achieved through work. Blum and Naylor [13] also define job satisfaction as the result of various attitudes employees hold towards their job; remuneration, level of supervision, the security of employment, the opportunity for career advancement, recognition of effort, timely settlement of grievances and fair evaluation of work.

Some studies on job satisfaction [12, 14, 15] conducted across various professions attribute job satisfaction to two main underlying factors: (1) achieving self-actualization (the feeling of worthwhile accomplishment from their job) and (2) environmental/physical rewards. Health professionals feel a sense of self-actualization [16] when the workplace presents conditions that are favourable for professional and personal growth as well as recognition for their effort and seeing noticeable progress of patients’ health [17]. Physical rewards include competitive salaries, autonomy, job security, flexible scheduling and fringe benefits [18]. Satisfied workers generally perform better in their line of duty [19, 20] and are less likely to absent themselves from work. Dissatisfaction among workers has been associated with poor commitment [21], absenteeism [22], and low quality of work and increased staff turnover [23]. For instance, in a systematic review of job satisfaction among physicians, Van Ham et al. [24] reported that some factors which influenced dissatisfaction among doctors included low income, longer working hours, administrative load and poor recognition at the workplace. A study by Chen et al. [14] showed that most healthcare practitioners were moderately satisfied with salary, autonomy and their relationship with co-workers.

### Job satisfaction among opticians

Ghana has a population of over 30 million people, with more than 12% of the population aged over 60 years (and a population growth rate of about 2%) [25]. With increase in population growth, there is an expected increase in the ageing population and a corresponding increase in diseases associated with ageing [26–28]. The change in population structure thus places a demand on eyecare professionals, especially opticians, in addressing vision-related disorders associated with ageing (such as presbyopia) [28, 29]. Age-related eye diseases/disorders, such as presbyopia, will result in an increased need for vision/eye care services (such as fitting of various forms of eyeglasses) and thus an anticipated need for more opticians to address this growing concern.

There are about 215 licensed opticians practising in Ghana and currently registered with the Opticians Association of Ghana (OAG). The Government of Ghana (through the Ghana Health Service and the Ministry of Health) is the largest employer of opticians in Ghana. The Allied Health Professionals Council, Ghana, is the body responsible for regulating activities of allied health workers, including opticians, in the country. In Ghana, opticians form a vital part of the eyecare system and play a critical role in eyecare delivery. The job description of an optician, as stipulated by the Ghana Health Service, is to provide efficient and quality eye care services through dispensing and fitting of eyeglasses and optical devices/aids [30]. They supply, fit and adjust ophthalmic lenses and frames, and other vision aids prescribed by optometrists or ophthalmologists. The dispensing optician examines written prescriptions to determine the specifications of ophthalmic lenses and recommends appropriate and suitable eyeglass frames, lenses and lens coatings.

Although job satisfaction surveys and studies have been widely reported for employees in various health cadres, such as nurses [31–33], physicians [34, 35], and physicians’ assistants [36, 37], very few studies on job satisfaction have been conducted among eyecare professionals, [15] and more specifically, among opticians [38] (even as their role in the eyecare sector cannot be underestimated). They provide relevant advice on lifestyle and occupational visual needs, child care, contact lens fitting and aftercare, and low vision aids [30]. These essential eye health services are significant in achieving VISION 2020 (which is to eliminate avoidable visual impairment and blindness) globally and more particularly in developing countries such as Ghana [39, 40]. In Africa and Ghana, efforts have been made to assess motivation and level of job satisfaction among health professionals, especially general physicians and nurses. However, there is paucity of data on job satisfaction among opticians in Ghana. Additionally, the World Health Organization’s (WHO) “Global Strategy on Human Resources for Health: Workforce 2030” [41] aims to ensure a well-equipped health workforce that will contribute effectively and efficiently towards the attainment of the Sustainable Development Goals (SDGs). These health workforces include human resources for eye health such as opticians. Addressing the various job challenges of opticians is therefore crucial in the social, economic and health development of a country and the overall success of the SDGs. The objectives of this study were: (1) to determine the overall level of satisfaction among opticians in Ghana; and (2) to determine the factors that are associated with job satisfaction among opticians in Ghana. Findings from this study will provide relevant stakeholders with evidence on job satisfaction among opticians in the country and ensure appropriate labour legislation and/or policy revision to preserve the interests of opticians in Ghana. Furthermore, this study will form the bedrock for future job satisfaction studies among opticians in Ghana and provide a prominent record to initiate policies and programmes targeted at forestalling fortunes and preventing deterioration of people becoming opticians.

## Methods

### Study design and participants

This study was a cross-sectional survey conducted from January to April 2019 among registered and licensed opticians in Ghana. The study was conducted among opticians working in the 10 regions of Ghana, namely Ashanti, Brong Ahafo, Central, Eastern, Greater Accra, Northern, Volta, Upper East, Upper West and the Western regions (Ghana had 10 regions as at the commencement of this study, but has recently been increased to 16 regions).

As at the beginning of our study, the professional registry of the Opticians Association of Ghana (OAG) had 215 registered opticians who were all eligible for the study. However, we conducted a further review of the OAG’s database and excluded 20 opticians who had no or incomplete contact information/details. Thus, a total of 195 opticians met our inclusion criteria and were subsequently contacted via email or telephone call/message to participate in the study. Nonetheless, a total of 101 opticians responded to our study, giving a response rate of 51.8%.

### Ethical approval

This study was conducted in adherence to the declaration of Helsinki and was approved by the Committee on Human Research, Publication and Ethics (CHRPE) of the Kwame Nkrumah University of Science and Technology (KNUST), Kumasi, Ghana (CHRPE/AP/069/19). Permission to conduct the study was obtained from the OAG. The purpose of the study was clearly explained to the participants and an informed written consent was obtained from all participants. Each participant was assigned a special identification code to help ensure that their identity was protected, and all data capturing adhered to strict confidentiality. Participants were made aware that there were no risks associated with their participation and that the data obtained would only be used for the study. Participation was voluntary and any participant was at liberty to withdraw from the study at any point in time.

### Measures

The instrument employed for this study was a validated, structured questionnaire adapted from Paudel et al.[42]. The questionnaires were administered either through a face-to-face interview or via email (Google form) [43]. Two Research Assistants administered face-to-face interviews during on-site data collection whereas data from all online questionnaires were received and managed by the Principal Investigator. It consisted of two parts: the first part, comprising 24 questions, captured sociodemographic data such as age, sex, marital status, number of children, as well as information on practice setting, educational level, regional distribution of opticians, type of work institution, good work–life balance, motivation for choice of profession, first job appointment, duration before first job appointment, working hours per week, years of work experience, reason for choosing opticianry, routine task areas, opportunity to choose another career, and having own/partnered established private practice. Work–life balance refers to the evenly allocation of one's time and focus between working and family, social or leisure activities. The second part consisting of 15 items assessed 15 key factors of job satisfaction including salary; non-financial incentives; job responsibility; co-workers; job security; work hours; supervision; task variety; equipment and facilities; continuing education; workload; control over work pace; support from co-workers, opportunities for career advancement; recognition; work acknowledgement; and participants overall perception of satisfaction (15^th^ item on the questionnaire). Each item in this section was scored on a five-point Likert scale; where 1 represented ‘very dissatisfied’, 2 being ‘dissatisfied’, 3 = ‘neither satisfied nor dissatisfied’, 4 = ‘satisfied’, and 5 being ‘very satisfied’. The score of each item and the mean score in each dimension were used in the analysis.

### Data analysis

The data were analysed using Statistical Product and Service Solution (IBM Corporation IBM® SPSS® Statistics for Windows, Version 25.0 Armonk, NY) compatible with Windows 10. Descriptive statistical analysis was used to summarize the sociodemographic factors of the participants. The mean score was calculated for all factors of job satisfaction. Job satisfaction of the opticians was further categorized into two groups: *satisfied* and *not satisfied*. Individuals with a score higher than 3 were placed in the *satisfied* group, and individuals with a score of 3 or lower were placed in the *not satisfied* group. The association between sociodemographic characteristics and factors of job satisfaction, and the overall level of job satisfaction was analysed using univariate and multivariate logistic regression analyses. Odds ratio (OR) and 95% confidence interval (CI) were then calculated. Statistical significance was set at P < 0.05.

## Results

One hundred and ninety-five (195) questionnaires were distributed to opticians throughout the country. Of these, one hundred and one (101) were included for analysis, indicating a response rate of 51.8%. Sixty-five opticians responded to the online questionnaires while 36 completed the printed questionnaires.

### Sociodemographic distribution

The mean (± SD) age of all participants was 25.3 ± 5.0 years (range: 21 to 47 years). Majority of the participants were males (n = 58; 57%), aged 30 years or less (n = 75; 74.3%), and worked in an urban setting (n = 74; 73.3%). Most respondents’ highest educational level was a Certificate in Dispensing Optics (n = 70; 69.3%). About three out of every four (n = 75; 74.3%) opticians reported that they had good work–life balance. Of note, 44.6% (n = 45) of the sampled opticians had their first job appointment more than 1 year after completing school. Table 1 represents the demographic characteristics of opticians enrolled in the study.

**Table 1:** Sociodemographic characteristics of participants

Characteristic	n (%)
Sex	
Male	58 (57.4)
Female	43 (42.6)
Age (years)	
≤ 30	75 (74.3)
31–45	25 (24.8)
46–60	1 (1.0)
Marital status	
Single	66 (65.3)
Married	35 (34.7)
Number of children	
0	66 (65.3)
≥ 1	35 (34.7)
Highest educational level	
Certificate in dispensing optics	70 (69.3)
Bachelor's degree (opticianry)	2 (2.0)
Diploma in dispensing optics	16 (15.8)
Post-graduate diploma (opticianry)	5 (5.0)
Master’s degree	1 (1.0)
Others	6 (5.9)
Regional distribution	
Ashanti	18 (17.8)
Brong Ahafo	6 (5.9)
Central	2 (2.0)
Eastern	10 (9.9)
Greater Accra	32 (31.7)
Northern	9 (8.9)
Volta	9 (8.9)
Western	6 (5.9)
Upper East	5 (5.0)
Upper West	4 (4.0)
Practice setting	
Urban	74 (73.3)
Rural	27 (26.7)
Type of institution	
Government	57 (56.4)
CHAG/NGO	26 (25.7)
Private	18 (17.8)
Working hours per week	
0–40	76 (75.2)
≥ 41	25 (24.8)
Work experience (years)	
0–5	79 (78.2)
6–10	12 (11.9)
≥ 11	10 (9.9)
Good work–life balance	
Yes	75 (74.3)
No	26 (25.7)
First job appointment	
Yes	79 (78.2)
No	22 (21.8)
Duration before first job appointment	
Within 3 months	37 (36.6)
3–6 months	9 (8.9)
6–12 months	10 (9.9)
> 1 year	45 (44.6)
*Reason for choosing opticianry	
Only available opportunity	12 (7.9)
Influence of family	3 (2.0)
Interest in eye care	91 (59.9)
Previous work experience with NGO	26 (17.0)
To earn good income	15 (9.9)
Did not know what to do after school	3 (2.0)
Other	2 (1.3)
*Routine task areas	
Optical dispensing	97 (96.0)
Refraction	49 (48.5)
Contact lens fitting	15 (14.9)
Community outreaches	65 (64.4)
Research activities	11 (10.9)
Diagnostic unit	16 (15.8)
Clinical examination	20 (19.8)
Given the chance would you choose another career	
Yes	29 (28.7)
No	72 (71.3)
Do you own/partnered an established private practice?	
Yes	14 (13.9)
No	87 (86.1)

n (%) represents the frequencies and percentages. *n ≠ 101 (results from multiple responses)

### Distribution of opticians in Ghana

In our study, we found majority of opticians to be working in the Greater Accra (n = 32; 31.7%) and Ashanti (n = 18; 17.8%) regions of Ghana. However, very few opticians worked in the Upper West (n = 4; 4.0%) and Upper East (n = 5; 5.0%) regions of Ghana (the northernmost part of the country). A total of 56.4% (n = 57) of opticians worked in government/public institutions as opposed to 17.8% (n = 18) of them in the private sector of the country. As many as 78.2% (n = 79) of the opticians had been working in their first job appointment. Regarding clinical practice, we found that almost all opticians in Ghana (n = 97; 96.0%) routinely dispensed optical corrections. However, about half of them (n = 49; 48.5%) performed refraction whereas more than 15.8% (n = 16) worked in diagnostic units. Interestingly, almost one in five (n = 20; 19.8%) opticians performed clinical examination, management and referrals (see Table 1).

### Level of job satisfaction among opticians in Ghana

Table 2 shows the degree of job satisfaction of all participants. The mean score (± SD) for overall perception of job satisfaction was 2.65 ± 0.74, with only 12.9% (95% CI: 7.0–21.0%) of opticians reporting satisfaction with their current jobs. The participants reported job satisfaction levels with regards to salary (3.30 ± 1.21), non-financial incentives (3.21 ± 1.13) and opportunity for continuous education (3.22 ± 1.22). However, less than half of the opticians (n = 49; 48.5%) reported that they were satisfied with their salary. Also, 66.3% (n = 67) participants reported that they were not satisfied with the variety in the tasks delegated to them. A total of 64.4% (n = 65) and 66.3% (n = 67) of the opticians reported that they were not satisfied with their level of supervision (2.88 ± 1.36), and support from co-workers (2.79 ± 1.30), respectively.

**Table 2:** Scores and percentage of key factors of job satisfaction and overall job satisfaction

Factors	Scores		
	Mean ± SD	Satisfied (%)	Not satisfied (%)
Salary	3.30 ± 1.21	48.5	51.5
Non-financial incentive	3.21 ± 1.13	40.5	59.5
Job security	3.14 ± 1.25	43.6	56.4
Workplace equipment	3.02 ± 1.41	42.6	57.4
Supervision	2.88 ± 1.36	35.6	64.4
Encouragement and feedback	3.00 ± 1.23	38.6	61.4
Recognition of work	2.94 ± 1.33	40.6	59.4
Level of responsibility	2.93 ± 1.24	37.6	62.4
Task variety	2.99 ± 1.10	33.7	66.3
Workload	3.14 ± 1.15	41.6	58.4
Level of control	2.79 ± 1.30	35.6	64.4
Support from co-workers	2.81 ± 1.19	33.7	66.3
Continuing education opportunities	3.22 ± 1.22	40.6	59.4
Career advancement opportunities	3.17 ± 1.21	41.6	58.4
Overall perception of job satisfaction	2.65 ± 0.74	12.9	87.1

“Overall perception of job satisfaction” represents the average score of the 14 factors associated with job satisfaction among participants

### Factors associated with job satisfaction among opticians in Ghana

Univariate logistic regression analysis, assessing factors associated with job satisfaction among opticians in Ghana, is shown in Table 3. There was no statistically significant association between all sociodemographic characteristics and overall level of job satisfaction (p > 0.05; for all). With reference to factors of job satisfaction among opticians in Ghana, only salary was significantly associated with overall level of job satisfaction (OR: 16.5; 95% CI: 2.06–132.86; p = 0.008). Therefore, there was no need for any model fitting and multiple logistic regression analysis in our study (given that only one variable [salary] was found to be statistically significantly associated with overall job satisfaction).

**Table 3:** Univariate logistic regression assessing factors associated with job satisfaction among opticians in Ghana

Characteristic	OR	95% CI	p-value
*Demographics*			
Age (years)	0.87	0.73–1.03	0.101
Sex			
Male	Ref		
Female	1.69	0.52–5.43	0.382
Marital status			
Single	Ref		
Married	0.53	0.14–2.05	0.354
Number of children	0.73	0.35–1.54	0.409
Location of workplace			
Urban	Ref		
Rural	1.26	0.35–4.47	0.725
Practice setting			
Government	Ref		
CHAG/NGO	1.30	0.35–4.90	0.699
Private	0.89	0.17–4.74	0.894
Working hours per week	1.04	0.94–1.15	0.471
Work experience (years)	0.87	0.70–1.09	0.237
Good work–life balance	4.76	0.59–38.57	0.144
*Factors for job satisfaction*			
Salary	16.5	2.06–132.86	0.008
Non-financial incentives			0.997
Job security			0.997
Workplace equipment			0.997
Supervision			0.997
Encouragement			0.997
Recognition			0.997
Responsibility to work			0.997
Task variety			0.997
Workload			0.997
Control			0.997
Support from co-workers			0.997
Continuing education opportunities			0.997
Career advancement opportunities			0.997

*OR* odds ratio, *CI* confidence intervals, *Ref* reference group; *p* ˂ 0.05 is considered statistically significant

## Discussion

This study presents new insights on the level of job satisfaction among opticians working in Ghana and its associated factors. Our study found that a little over a tenth (12.9%) of opticians working in Ghana were satisfied with their jobs. Sociodemographic characteristics (including age, sex, marital status, practice setting, educational level, number of working hours) were not significantly associated with overall level of job satisfaction. However, salary was significantly associated with overall level of job satisfaction among opticians in Ghana.

Job satisfaction surveys have been studied in many countries and across several professions. In this study, we found that minority (12.9%; 95% CI: 7.0–21.0%) of opticians working in Ghana were satisfied with their jobs. This was similar to findings from a study among healthcare practitioners in Ethiopia [44], which reported a low level of overall job satisfaction. However, in the optical workforce survey conducted in the United Kingdom, majority of opticians (79%) were satisfied with their jobs. The low level of satisfaction recorded among Ghanaian opticians could be driven by economic factors such as bonuses, pay raise and social factors like supportive work environment. In terms of the disparity in job satisfaction relative to other studies, a plausible explanation for this trend could be the nature of the primary health care system in Ghana. In developed countries, eyecare professionals operate a work-based appointment system. Thus, they generally have a well laid-out schedule and are able to anticipate how the day turns out [14], as opposed to the Ghanaian health setting which operates less organized walk-in services.

We found no statistically significant association between sociodemographic variables and the level of job satisfaction in our study. Similar findings by Deriba et al. [2] and Van Ham et al. [24] also showed that job satisfaction was not influenced by demographic characteristics. The lack of a clear trend between job satisfaction and sociodemographic factors could be attributed to the fact that most opticians receive similar remuneration and service benefits regardless of age, gender, marital status, or the number of years in practice. Contrary to our findings, some studies [45, 46] have reported that factors such as marital status [45], hospital location [47], and number of years in practice [48] had influence on job satisfaction. The reason being that employees with the highest professional achievement have more autonomy [49], flexible schedules, occupy positions that come with less stress [50], and are more likely to benefit from travel and continuing education opportunities.

From our study, salary (OR: 16.5; 95% CI: 2.06–132.86) was found to be the only factor significantly associated with overall job satisfaction among opticians in Ghana. A systematic review on job satisfaction among general medical practitioners showed that inadequate income accounted for low level of satisfaction [24]. Salaries are seen as a recompense for time and effort expended and are a perceived measure of their value as professionals. According to Herzberg’s theory of motivation [7], employees are more satisfied with their jobs if they have a competitive salary, attractive and appealing reward system. Such employees are likely to conduct their duties with enthusiasm. Salary is generally perceived to be quite low among healthcare professionals in Ghana [51]. Lower remuneration to these healthcare professionals, such as opticians, may result in low work output or productivity [23]. Thus, stakeholders (public and private) in Ghana’s ophthalmic industry need to adopt policies aimed at enhancing salary levels and job motivation among opticians.

This study reported a combined response rate of 51.8% for the completed questionnaires (online and printed). In most job satisfaction surveys conducted among health professionals, response rates usually vary between 30 to 90% [24, 38, 42, 45, 48]. Surveys conducted in single sectors had higher response rates because it was easier to reach all respondents at a time while cross-sectional and multi-centre studies had moderate to low response rate. In Ghana, combined response rates for surveys among Ghanaian eyecare professionals, using both online and printed questionnaires, are 33.5% [52] and 46% [53]. Although the response rate in our study was relatively higher as compared to similar surveys among eyecare professionals in Ghana, the busy schedule of opticians in their ophthalmic settings, constraints in follow-up, inadequate resources, unstable internet networks, and other official engagements by some opticians made it impossible to achieve a much higher response rate in our study.

The strength of this study was that it captured data of opticians in all 10 regions of Ghana. Another strength of this study was that we used a questionnaire, which had been validated for use among eyecare professionals in other countries, and thus provides reliable data regarding opticians’ perception of their job satisfaction level. However, our study could not establish the causative relationship between job satisfaction level and its associated factors due to the cross-sectional nature/design used in our study. Future studies must be conducted to assess the association between occupational safety, social relations within eyecare institutions, communication, and job satisfaction among opticians in Ghana. A qualitative study design may also be employed in future studies to examine opticians’ behaviour, opinions and motivations in relation to their level of job satisfaction in Ghana.

## Conclusion

In conclusion, we found out that only about one in ten opticians working in Ghana were satisfied with their current jobs. Salary was significantly associated with overall job satisfaction level. The novel findings from this study would assist relevant stakeholders, policymakers, and health managers to effectively manage human resources for eye-health (especially opticians) and revise policy for ophthalmic healthcare administration in Ghana.

## Data Availability

The dataset(s) supporting the conclusions of this article is/are available on request from the corresponding author.

## Abbreviations

ERG: Existence, Relatedness and Growth theory
O.A.G.: Opticians Association of Ghana.
S.D.G.: Sustainable Development Goals
W.H.O.: World Health Organization

## References

[1] Mosadeghrad AM (2014). Factors influencing healthcare service quality. Int J Health Policy Manag.

[2] Deriba BK, Sinke SO, Ereso BM, Badacho AS (2017). Health professionals' job satisfaction and associated factors at public health centers in West Ethiopia. Hum Resour Health.

[3] Bagheri S, Kousha A, Janati A, Asghari-Jafarabadi M (2012). Factors influencing the job satisfaction of health system employees in tabriz, iran. Health promotion perspectives.

[4] Van der Vaart L, Linde B, De Beer L, Cockeran M (2015). Employee well-being, intention to leave and perceived employability: a psychological contract approach. South Afr J Econ Manag Sci.

[5] Oliveira MZ, Natividade JC, Assis RS, Mambrini NSB (2019). Performance, satisfaction and intention to remain in organizations: individual to contextual predictors. Trends Psychol.

[6] Maslow A, Lewis K (1987). Maslow's hierarchy of needs. Salenger Incorporated.

[7] Herzberg F (1965). The motivation to work: two factor theory. Pers Psychol.

[8] Alderfer CP (1969). An empirical test of a new theory of human needs. Organ Behav Hum Perform.

[9] Vroom V, Porter L, Lawler E (1964). Expectancy theories. Organ Behav.

[10] Lawler EE, Suttle JL (1973). Expectancy Theory and Job Behaviour. Organiz Behav Human Perf.

[11] Locke EA, Latham GP: Goal setting theory. In: *Motivation: Theory and research. Volume 1*, edn.: Routledge; 2012: 23–40.

[12] Sinha D: Job satisfaction and job behaviour. Motivation and Organizational Effectiveness New Delhi: Shri Ram Centre for Industrial Relations and Human Resources. 1972;136–174.

[13] Blum ML, Naylor JC: Industrial psychology; Its theoretical and social foundations. Harper & Row; 1968, p. 1–633.

[14] Chen AH, Jaafar SN, Noor AR (2012). Comparison of job satisfaction among eight health care professions in private (non-government) settings. Malays J Med Sci.

[15] Khachatryan N, Bowen M, Cordiner M, Melkonyan A (2012). The UK Optometric Workforce Survey: job satisfaction among optometrists. Invest Ophthalmol Vis Sci.

[16] Hartzell S: The Needs Theory: Motivating Employees with Maslow's Hierarchy of Needs. 2014.

[17] Sinsky CA, Willard-Grace R, Schutzbank AM, Sinsky TA, Margolius D, Bodenheimer T (2013). In search of joy in practice: a report of 23 high-functioning primary care practices. Ann Fam Med.

[18] Iqbal S, Guohao L, Akhtar S (2017). Effects of job organizational culture, benefits, salary on job satisfaction ultimately affecting employee retention. Review Pub Admin Manag.

[19] Wright TA, Cropanzano R, Bonett DG (2007). The moderating role of employee positive well-being on the relation between job satisfaction and job performance. J Occup Health Psychol.

[20] Ajayi MP, Abimbola OH (2013). Job satisfaction, organizational stress and employee performance: A study of NAPIMS. IFE PsychologIA.

[21] Vance RJ: Employee Engagement and Commitment: A guide to understanding, Measuring and Increasing Engagement in your Organization., vol. 1. Duke Street, Alexandria.: Society for Human Resource Management Foundation; 2006.

[22] Oche M, Oladigbolu R, Ango J, Okafoagu N, Ango U (2018). Work absenteeism amongst health care workers in a tertiary health institution in Sokoto, Nigeria. J Adv Med Med Res.

[23] Aduo-Adjei K, Emmanuel O, Forster OM (2016). The impact of motivation on the work performance of health workers (Korle Bu Teaching Hospital): Evidence from Ghana. Hosp Pract Res.

[24] Van Ham I, Verhoeven AAH, Groenier KH, Groothoff JW, De Haan J (2006). Job satisfaction among general practitioners: a systematic literature review. Eur J Gen Pract.

[25] Nation U, United Nations (2019). World Population Prospects 2019: Methodology of the United Nations Population Estimates and Projections. Population Division World Population Prospects United Nations.

[26] Akpek EK, Smith RA (2013). Overview of age-related ocular conditions. Am J Manag Care.

[27] Chen Y, Hahn P, Sloan FA (2016). Changes in Visual Function in the Elderly Population in the United States: 1995–2010. Ophthalmic Epidemiol.

[28] Salvi SM, Akhtar S, Currie Z (2006). Ageing changes in the eye. Postgrad Med J.

[29] Chader GJ, Taylor A (2013). Preface: the aging eye: normal changes, age-related diseases, and sight-saving approaches. Invest Ophthalmol Vis Sci.

[30] Service GH: Job Description for Allied Health Staff. In: *Job description of Optician.* Accra: Ministry of Health, Ghana; 2005: 1–183.

[31] Semachew A, Belachew T, Tesfaye T, Adinew YM (2017). Predictors of job satisfaction among nurses working in Ethiopian public hospitals, 2014: institution-based cross-sectional study. Hum Resour Health.

[32] Lu H, Zhao Y, While A (2005). Job satisfaction among hospital nurses: a literature review. Int J Nurs Stud.

[33] De la Fuente-Solana EI, Suleiman-Martos N, Pradas-Hernández L, Gomez-Urquiza JL, Albendín-García L (2019). Prevalence, related factors, and levels of burnout syndrome among nurses working in gynecology and obstetrics services: a systematic review and meta-analysis. Int J Environ Res Public Health.

[34] Sabitova A, McGranahan R, Altamore F, Jovanovic N, Windle E, Priebe S (2020). Indicators associated with job morale among physicians and dentists in low-income and middle-income countries: a systematic review and meta-analysis. JAMA Netw Open.

[35] Rosta J, Aasland OG, Nylenna M (2019). Changes in job satisfaction among doctors in Norway from 2010 to 2017: a study based on repeated surveys. BMJ Open.

[36] Hoff T, Carabetta S, Collinson GE (2019). Satisfaction, burnout, and turnover among nurse practitioners and physician assistants: a review of the empirical literature. Med Care Res Rev.

[37] Osborn M, Satrom J, Schlenker A, Hazel M, Mason M, Hartwig K (2019). Physician assistant burnout, job satisfaction, and career flexibility in Minnesota. J Am Acad PAs.

[38] College of Optometrists: The Optical Workforce Survey Full Report. In*.*, vol. 1, 2 edn. London, : The College of Optometrists; 2016: 1–73.

[39] Palmer JJ, Chinanayi F, Gilbert A, Pillay D, Fox S, Jaggernath J, Naidoo K, Graham R, Patel D, Blanchet K (2014). Trends and implications for achieving VISION 2020 human resources for eye health targets in 16 countries of sub-Saharan Africa by the year 2020. Hum Resour Health.

[40] Palmer JJ, Chinanayi F, Gilbert A, Pillay D, Fox S, Jaggernath J, Naidoo K, Graham R, Patel D, Blanchet K (2014). Mapping human resources for eye health in 21 countries of sub-Saharan Africa: current progress towards VISION 2020. Hum Resour Health.

[41] World Health Organization: Global Strategy on Human Resources for Health: Workforce 2030. In: *Workforce.* vol. 2016. Geneva, Switzerland: World Health Organization; 2016: 1- 64.

[42] Paudel P, Cronjé S, O'Connor PM, Khadka J, Rao GN, Holden BA (2017). Development and validation of an instrument to assess job satisfaction in eye-care personnel. Clin Exp Optom.

[43] Rayhan RU, Zheng Y, Uddin E, Timbol C, Adewuyi O, Baraniuk JN (2013). Administer and collect medical questionnaires with Google documents: a simple, safe, and free system. Appl Med Inform.

[44] Gedif G, Sisay Y, Alebel A, Belay YA (2018). Level of job satisfaction and associated factors among health care professionals working at University of Gondar Referral Hospital, Northwest Ethiopia: a cross-sectional study. BMC Res Notes.

[45] Al Khalidi D, Wazaify M (2013). Assessment of pharmacists' job satisfaction and job related stress in Amman. Int J Clin Pharm.

[46] Atif K, Khan HU, Maqbool S (2015). Job satisfaction among doctors, a multi-faceted subject studied at a tertiary care hospital in Lahore. Pakistan J Med Sci.

[47] Farmer J, Hinds K, Richards H, Godden D (2005). Urban versus rural populations' views of health care in Scotland. J Health Serv Res Policy.

[48] Lu Y, Hu XM, Huang XL, Zhuang XD, Guo P, Feng LF, Hu W, Chen L, Hao YT (2016). Job satisfaction and associated factors among healthcare staff: a cross-sectional study in Guangdong Province China. BMJ Open.

[49] Grossman HY, Chester NL: The experience and meaning of work in women's lives, vol. 1: Psychology Press; 2013.

[50] Graham KR, Davies BL, Woodend AK, Simpson J, Mantha SL (2011). Impacting Canadian public health nurses’ job satisfaction. Can J Public Health.

[51] Alhassan RK, Spieker N, van Ostenberg P, Ogink A, Nketiah-Amponsah E, de Wit TFR (2013). Association between health worker motivation and healthcare quality efforts in Ghana. Hum Resour Health.

[52] Asiedu K, Kyei S, Ayobi B, Agyemang FO, Ablordeppey RK (2016). Survey of eye practitioners’ preference of diagnostic tests and treatment modalities for dry eye in Ghana. Cont Lens Anterior Eye.

[53] Ocansey S, Abu EK, Nii Armah O, Morny EK: The practise of paediatric optometry in a low‐resource environment. *Clinical and Experimental Optometry* 2019. 10.1111/cxo.13005 31852024

